# High DNA Methylation Pattern Intratumoral Diversity Implies Weak Selection in Many Human Colorectal Cancers

**DOI:** 10.1371/journal.pone.0021657

**Published:** 2011-06-28

**Authors:** Kimberly D. Siegmund, Paul Marjoram, Simon Tavaré, Darryl Shibata

**Affiliations:** 1 Department of Preventive Medicine, University of Southern California Keck School of Medicine, Los Angeles, California, United States of America; 2 Department of Biological Sciences, University of Southern California, Los Angeles, California, United States of America; 3 Department of Oncology, University of Cambridge, Cambridge, United Kingdom; 4 Department of Pathology, University of Southern California Keck School of Medicine, Los Angeles, California, United States of America; University of Hong Kong, Hong Kong

## Abstract

**Background:**

It is possible to infer the past of populations by comparing genomes between individuals. In general, older populations have more genomic diversity than younger populations. The force of selection can also be inferred from population diversity. If selection is strong and frequently eliminates less fit variants, diversity will be limited because new, initially homogeneous populations constantly emerge.

**Methodology and Results:**

Here we translate a population genetics approach to human somatic cancer cell populations by measuring genomic diversity within and between small colorectal cancer (CRC) glands. Control tissue culture and xenograft experiments demonstrate that the population diversity of certain passenger DNA methylation patterns is reduced after cloning but subsequently increases with time. When measured in CRC gland populations, passenger methylation diversity from different parts of nine CRCs was relatively high and uniform, consistent with older, stable lineages rather than mixtures of younger homogeneous populations arising from frequent cycles of selection. The diversity of six metastases was also high, suggesting dissemination early after transformation. Diversity was lower in DNA mismatch repair deficient CRC glands, possibly suggesting more selection and the elimination of less fit variants when mutation rates are elevated.

**Conclusion/Significance:**

The many hitchhiking passenger variants observed in primary and metastatic CRC cell populations are consistent with relatively old populations, suggesting that clonal evolution leading to selective sweeps may be rare after transformation. Selection in human cancers appears to be a weaker than presumed force after transformation, consistent with the observed rarity of driver mutations in cancer genomes. Phenotypic plasticity rather than the stepwise acquisition of new driver mutations may better account for the many different phenotypes within human tumors.

## Introduction

**Figure 1:**
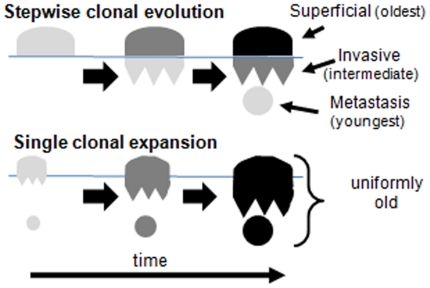
Two progression models. Stepwise selection and clonal evolution creates populations of different diversities and phenotypes because they are created at different times from different progenitors after transformation. By contrast, the diversity of a single clonal expansion is relatively uniform.

A barrier to a better understanding of human cancer is the inability to directly observe how cancers evolve. Progression is clinically important, with localized cancers more easily treated than deeply invasive or metastatic cancers. A logical presumption is that progression occurs stepwise after transformation (Fig 1) with the sequential selection of new driver mutations and more malignant phenotypes by clonal evolution [1] as tumor cells encounter and then colonize new microenvironments. However, a recent cancer genome sequencing study [2] illustrated that metastases have relatively few additional mutations compared to their primary colorectal cancers (CRCs). Approximately 97% of the mutations present in the metastases were also detected in their primary tumors, and the few additional mutations did not have obvious metastatic roles.

Alternatively, the ability to invade or metastasize may already be present at the time of transformation [3], allowing for progression without further clonal evolution (Fig 1). Phenotypic differences between tumor cells would arise secondary to phenotypic plasticity [4], [5] rather than the acquisition of new driver mutations. A key difference between these models is the efficiency of selection to act upon variant cells that inevitably accumulate with time. Selection depends on variation, but selection may not easily discriminate between cancer cells because most mutations appear to be neutral passenger mutations [6], [7]. Although positive or negative selection is difficult to quantify, there is a long history of using the variation at neutral or hitchhiking passenger loci within a population to measure the force of selection, which opposes drift by eliminating less fit variants [8]. Because neutral passenger changes are more common than driver changes [6], [7], many passenger changes may accumulate within tumor populations between selective sweeps.

Tumor heterogeneity measurements are complicated because many mechanisms can contribute to diversity [4]. Measuring the diversity in a large tumor population is problematic because if selection is strong, many different variants may be selected, each optimal for survival within the many different microenvironments of a tumor. To minimize environmental heterogeneity and because the most immediate battle for survival occurs between adjacent cells, a sensitive test for selection is the amount of variation among small groups of neighboring cells within a single microenvironment, especially since fixation is faster in smaller populations [9]. Although progeny of a single selected cell may not sweep widely, at a minimum selection should be able to homogenize its immediate neighborhood and microenvironment. Extensive heterogeneity between adjacent cancer cells would suggest selection does not frequently optimize fitness even within small populations.

**Figure 2:**
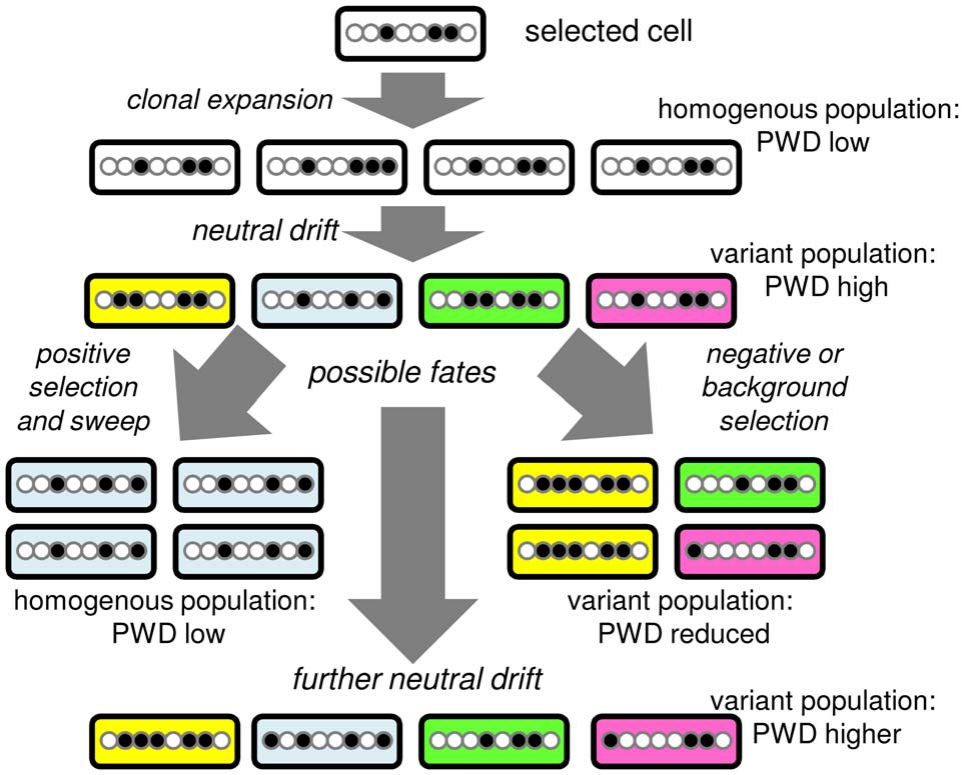
Hitchhiking neutral or passenger methylation. A male cancer cell contains a single methylation pattern on a CpG rich region of the X-chromosome. The passenger methylation pattern will drift and hitchhike with the fate of its cell. After clonal expansion, the cancer cell population will be initially homogeneous but variant cells (different colors) and passenger methylation patterns will arise from drift (replication errors). A diverse population has three fates. If no selection occurs, a population will continue to drift and become more diverse. Strong positive selection of a variant (blue) cell results in a sweep or clonal evolution, with homogenization of the population and hitchhiking passenger methylation. Weak negative or background selection leads to loss of a variant cell (blue) and a reduction in the diversity of the hitchhiking methylation patterns. Therefore, the strength of selection can be inferred by measuring the PWDs of hitchhiking passenger methylation patterns within a population.

Colorectal adenocarcinomas have neoplastic glands that partition tumor cells into distinct small neighborhoods. The extent of hitchhiking diversity within and between glands depends on the timing of the last clonal expansion or selective sweep (Fig 2). A selected variant cell and its progeny would initially dominate its gland and could subsequently form additional neighboring glands, creating a focal population with relatively uniform diversity. At its extreme, an entire tumor may be created by a single clonal expansion. Here we measure cancer genome passenger DNA methylation pattern variation within small (2,000 to 10,000 cell) gland fragments from different parts of the same human CRCs and infer clonal evolutionary bottlenecks occur infrequently after transformation.

## Results

### Detecting Selection and Clonal Evolution in an Experimental System

**Figure 3:**
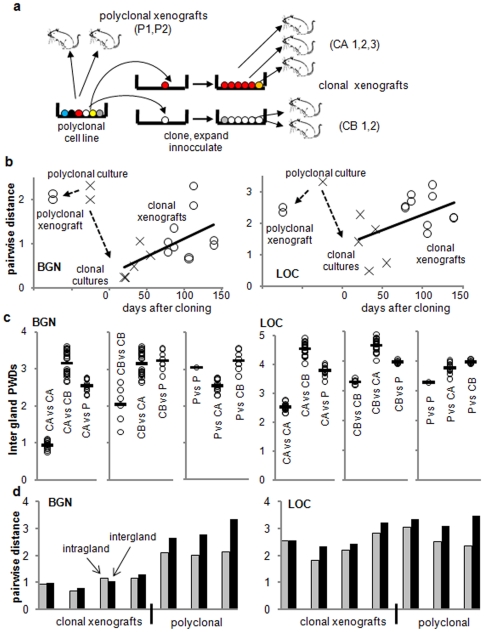
Experimental clonal evolution. **A.** Schematic of the Lovo single cell cloning, first in culture and then as xenografts, simulating a clonal evolution bottleneck. **B.** Hitchhiking diversity decreases and then increases in culture and the xenografts after single cell cloning. (X's represent independent single cell clones in culture, and O's represent PWD averages among tags isolated from 5 to 6 small xenograft fragments) The LOC tag has a higher error rate compared to the BGN tag [12], and more quickly restores the diversity seen in polyclonal populations. **C.** Comparisons between fragments demonstrate intergland PWDs are smaller between clonally related tumors and larger between unrelated tumors, indicating the ability of the LOC or BGN tags to identify and distinguish between new and older clonal expansions (circles are averages of the fragment comparisons between the different xenografts, and bars are overall averages) **D.** Xenograft intragland PWDs are typically nearly as large as their intergland PWDs, indicating that the small tumor fragments are almost as diverse as their tumors.

Because somatic mutations are relatively rare in human CRCs (<1 per 100,000 bases [6], [10]), we have employed the 5′ to 3′ order of passenger DNA methylation at short CpG rich regions as epigenetic somatic cell molecular clocks [11]. The 5′ to 3′ order of methylation is used (like the 5′ to 3′ order of base sequences) to measure genomic variation, which should hitchhike with the fates of their cells (Fig 2). Passenger methylation patterns should be initially homogeneous and subsequently become increasingly polymorphic after a clonal evolution bottleneck. In the first experiments, we verify that these passenger methylation patterns or tags can record a simple clonal evolution cycle: polyclonal population → monoclonal population → polyclonal population. This bottleneck (Fig 2) can be simulated by cloning and then expanding single cells in tissue culture and subsequently as subcutaneous xenografts in nude mice (Fig 3A). X-chromosome tags (BGN and LOC, tag sequences and sample data are provided in Fig S1) and a male diploid CRC cell line (Lovo) were examined (a single epiallele per cell). The LOC tag appears to have a higher replication error rate than the BGN tag [12], and therefore should become polymorphic faster. Epialleles were sampled by bisulfite sequencing cloned PCR products of DNA extracted from the cultures or small xenograft fragments (5 to 6 fragments per xenograft, ∼2,000 to 10,000 cells per fragment). Diversity is measured by a pairwise distance (PWD).

The polyclonal culture and small fragments of its xenografts were diverse populations, with many different tag patterns and relatively high PWDs (Fig 3). After single cell cloning, tag diversity decreased but subsequently increased. This reduction and subsequent increase in tag diversity after single cell cloning was not strictly clock-like because some younger clones were more diverse than some older clones (Fig 3B). However, tag pattern changes generally recorded the experimental clonal evolution scenario of a single cell bottleneck followed by clonal expansion and an increase in PWDs.

### Detecting Stepwise Progression In An Experimental System

The above studies measured diversity within single tumor gland fragments. Another method to measure diversity is to compare epialleles from different parts of the same tumor. Here PWDs between cells are compared with physical distances between cells to ask whether adjacent cells are more related than distant cells. With a single rapid clonal expansion (a star phylogeny), distant cells are almost related as adjacent cells because its different parts are essentially created at the same time. Therefore, PWDs will be independent of physical distance or location. By contrast, if tumors are created by stepwise selection and clonal evolution, PWDs depend on the physical location of the cells. Older regions should be more diverse than younger regions, and PWDs should increase when comparisons are made between younger and older tumor regions (Fig 1).

The xenografts simulate these different progression scenarios. A single recent clonal expansion is represented by xenografts initiated from the same single cell progenitor. Stepwise clonal evolution is represented by comparisons between the polyclonal and clonal xenografts. Intergland PWDs were lower in the clonal xenografts, greater in the polyclonal xenografts, and greater between the clonal xenografts and their parental polyclonal xenografts or the other independent clonal xenograft (Fig 3C). These experiments demonstrate that passenger methylation tags can distinguish a single clonal expansion from tumors composed of different aged populations.

### Lack of Bottlenecks During Xenograft Formation

The cloning experiments are potentially complicated by unseen bottlenecks caused by natural selection (Fig 2). The “polyclonal” nature of the Lovo cell line in tissue culture before single cell cloning may not be unexpected because this is a long established cell line [13], and presumably progeny are similarly fit. However, bottlenecks may occur during xenograft formation because only some cells within a tissue culture adapted cell line may thrive in a nude mouse microenvironment. Indeed, xenograft growth may not be visible when less than a million cells are inoculated, a phenomenon often employed to measure frequencies of “cancer initiating cells”, which may represent cancer stem cells (CSCs) [14]. Bottlenecks may also occur if different microenvironments encountered during growth efficiently select only the fittest variants, to yield localized clonal evolution.

If significant bottlenecks occur during tumorigenesis, xenograft diversities will be similarly limited whether the initial inoculate was clonal or polyclonal. BGN tag diversity was significant less when xenografts were initiated with the single cell clones compared with a polyclonal inoculate (average intragland PWDs of 1.2 versus 2.1, p = 0.007), indicating that selective bottlenecks do not occur with the Lovo cell line during xenograft tumorigenesis (Fig 3B). LOC tag diversity was similar between clonal and polyclonal xenograft fragments (average intragland PWDs of 2.5 versus 2.4, p = 0.86), but the LOC tag appears to have a higher replication error rate than BGN [12]. Therefore the similarities in LOC tag diversity appear to result from the more rapid LOC tag diversification with the single cell clones rather than tumorigenesis bottlenecks.

Another way to detect localized selection is to compare the diversity within glands with the diversity between glands. In the absence of selection, older more diverse tumors should have older more diverse glands. If selection occurs within glands, diversity is reduced as variant cells are eliminated (Fig 2), and intragland PWDs should be much smaller than intergland PWDs. Consistent with a lack of selection, intragland PWDs were nearly as large as intergland PWDs for the clonal and polyclonal xenografts (Fig 3D).

### Deeper Sampling Confirms High Passenger Tag Diversity

**Figure 4:**
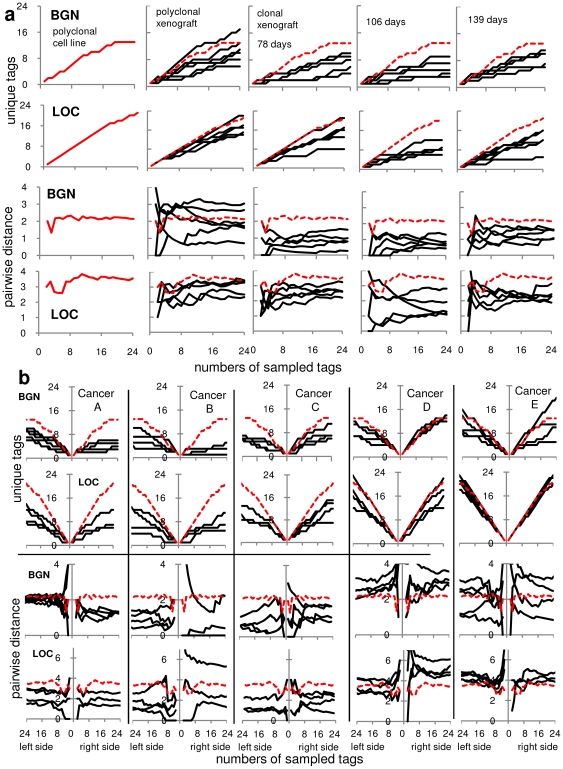
Deeper sampling. **A.** More unique epiallele patterns are observed within polyclonal cultures and the small xenograft fragments when sampling is increased to 24 tags. PWD values are relatively stable after 8 sampled tags (for reference, the dotted red lines are the polyclonal cell line values). **B.** Deeper sampling of glands from five human CRCs. The three cancers on the left are relatively younger cancers with diversity similar to the clonal xenografts. The two cancers on the right are relatively older cancers with diversity similar to the polyclonal cell line or xenografts. Consistent with a single clonal expansion, diversity is similar between right and left parts of the same CRC.

In the above studies, only eight tags were sampled per specimen. To confirm the diversity in the small fragments, 24 tags were sampled (Fig 4A). Additional new tag patterns were detected with further sampling, although PWDs were relatively stable after sampling only eight tags. Average diversity was higher in the polyclonal versus the clonal xenografts, with averages of 10.8 BGN and 17.2 LOC unique patterns per 24 sampled tags in the polyclonal xenografts. Substantial tag variation is present in small 2,000 to 10,000 cell tumor fragments, further suggesting that selection leading to clonal evolution rarely occurs during xenograft formation with the Lovo cell line in a nude mouse environment.

### High Diversity in Single Human Colorectal Cancer Glands

A prior study inferred that small human CRC glands are diverse populations [12], but sampled only eight tags per gland. To confirm that human CRC gland diversity may be high and allow comparisons with the xenografts, 24 tags were sampled from 2,000 to 10,000 cell gland fragments from five CRCs. The glands were sampled from opposite sides (“left” and “right”) of the same tumor, to allow comparisons of cells located within glands and between glands from opposite tumor sides.

Human CRC gland diversities were high (between 3 to 23 unique patterns per 24 sampled epialleles) indicating that neighboring cells are not closely related (Fig 4B). For three less diverse (“younger”) cancers (Cancers A,B,C), gland PWDs and numbers of unique tags per 24 sampled tags were similar to values in the clonal xenografts. For two more diverse (“older”) cancers (Cancers D,E), gland PWDs and numbers of unique tags were similar to the values in the polyclonal xenograft. Consistent with a single clonal expansion, diversities were similar between left and right tumor sides, indicating that both tumor sides are likely to have similar mitotic ages or numbers of divisions since transformation [12].

### Lower Gland Diversity With Higher Mutation Rates

**Figure 5:**
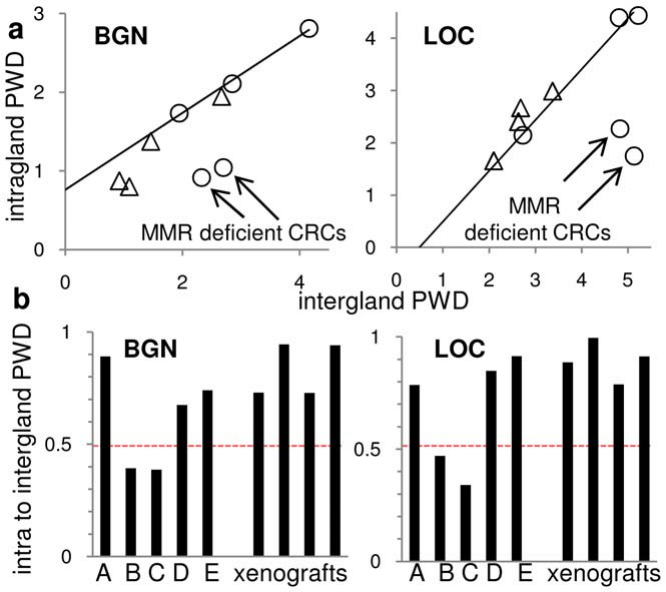
Intragland versus intergland PWDs. **A.** Intra- and intergland PWDs generally correlate. The trend line is based on three human CRCs, with the two MMR deficient cancers with much lower intragland PWDs. (circles are human CRCs, triangles are the clonal and polyclonal xenografts). **B.** Ratios of intra- to intergland PWDs were greater than 0.5 except for the MMR deficient CRCs, indicating much greater extinction within glands of the MMR deficient CRCs.

Low cancer gland diversity does not directly reflect selection because even without selection a new clonal expansion would initially be homogeneous. To correct for tumor age, as a rough approximation, the age of a tumor is the time or numbers of divisions between cells from opposite tumor sides, because these cells last shared a common ancestor around the time of transformation [12], [15]. Therefore, one can compare gland age with tumor age by comparing intragland PWDs with PWDs between glands from opposite cancer sides (Fig 5A). In general, older tumors had older glands, with intragland PWDs correlated with intergland PWDs.

If selection depends on mutations, a simple prediction is that selection should occur more often when mutation rates are elevated. Two of the five CRCs (Cancers B,C) were deficient in DNA mismatch repair (MMR), which is associated with 100- to 1000-fold higher mutation rates [16]. Gland PWDs in the MMR deficient CRCs were lower (intragland to intergland PWDs less than 50%) relative to the MMR proficient CRCs (Fig 5B). The lower intragland to intergland PWD ratios in MMR deficient CRCs suggests selection may more efficiently eliminate less fit variants when mutation rates are higher. The intragland to intergland PWD ratios for the three MMR proficient CRCs and the Lovo xenografts were similar (greater than 50%) suggesting selection occurs less often in these tumors.

### Cancer Stem Cells Appear To Be Frequent in Human CRCs

A quantitative way to describe selection is to estimate the numbers of long-lived lineages per cancer gland, which are effectively CSCs [12]. If selection or extinction occurs frequently, then long-lived CSC lineages will be few. The number of CSCs per cancer gland can be estimated from its diversity---genomes within a gland will be more similar with smaller numbers of CSCs because somatic alterations cannot accumulate in shorter lived non-CSCs that rapidly become extinct.

A previous analysis of data with eight tags per gland estimated relatively high CSC frequencies [12]. With 24 tags per gland, better estimates of CSC frequencies are possible (Table 1). Cancer gland diversity was too high for a single CSC per gland and too low for a scenario in which no extinction occurs. Instead, cancer gland variation was more consistent with limited cancer cell extinction, which can be modeled as a stem cell hierarchy with multiple CSCs per gland producing a limited number of non-CSC progeny. Probabilistic CSC survival instead of deterministic asymmetric CSC division was more consistent with the data. Estimated numbers of probabilistic CSCs per 8,000 cell gland were between 128 and 2,048. The lowest estimated CSC frequencies per gland (128 and 256 per gland) were with the two MMR deficient Cancers B and C, a trend previously noted when comparing between MMR proficient and deficient CRCs [12]. In summary, cancer cell extinction appears to have occurred in all the CRCs, but more extensively in MMR deficient CRC glands.

**Table 1:** Estimated numbers of CSCs per 8,000 cell cancer gland.

Cancer	MMR deficient	estimated CSC per gland	experimental values within 95% simulation intervals			
			1 CSC per gland*	all CSCs*	multiple, immortal*	multiple, random*
A	No	512	1/34	16/34	25/34 (8)	32/34 (512)
B	Yes	128	5/28	3/28	14/28 (4)	22/28 (128)
C	Yes	256	0/30	3/30	17/30 (4)	26/30 (256)
D	No	512	0/32	15/32	19/32 (16)	26/32 (512)
E	No	2048	0/34	22/34	21/34 (32)	26/34 (2048)

*Numbers of experimental values (PWDs within glands or unique tags per 24 sampled tags) within 95% simulation intervals for the four scenarios. Simulated CSC numbers (multiples of 2) best fitting the multiple immortal or random CSC per gland scenarios are in parentheses.

### Clonal Evolution In Human Tumor Populations

**Figure 6:**
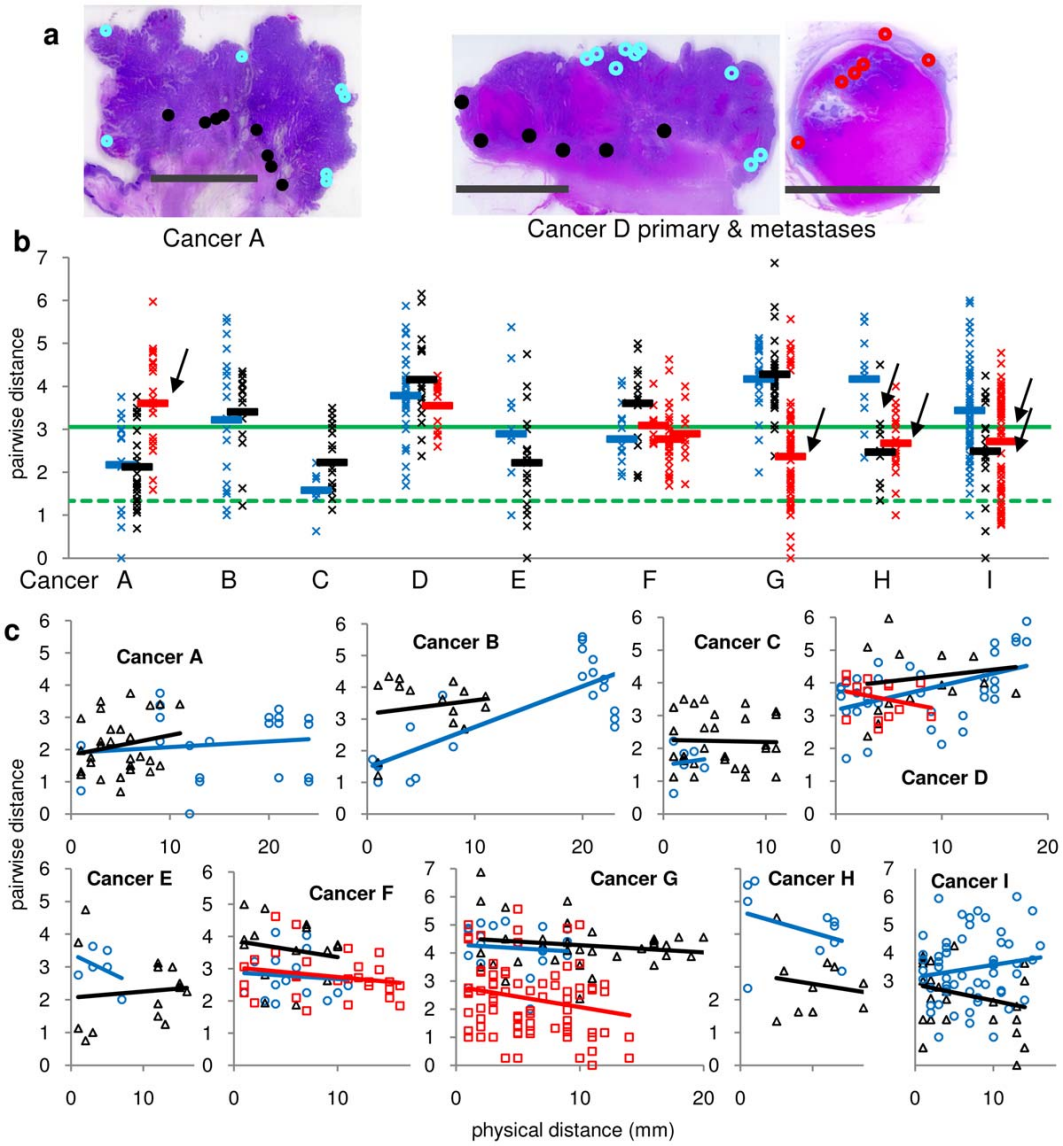
Diversity in human invasive and metastatic CRCs. **A.** Diagram of the LCM sampling of Cancers A and D. Dots are locations of the superficial (blue), invasive (black), and metastatic (red) regions. (bar is 1 cm wide). **B.** Comparison of intergland PWDs in the superficial (blue), invasive (black) and metastatic (red) regions of the nine CRCs. PWDs between individual LCM samples (“X”) are scattered, with averages represented by the bars. The scatter of the PWDs between individual glands is expected because of the stochastic nature of replication errors, and regions within the same tumor that were significantly different (arrows) from their superficial regions were identified from simulations (see Methods). For reference, the intergland PWDs of the clonal (dotted green line) and polyclonal xenografts (solid green line) are illustrated. **C.** Comparisons of intergland PWDs with physical distances indicate that distant and adjacent glands are similarly related in the superficial (blue), invasive (black) and metastatic (red) regions. A significant increase in PWDs with physical distance (p<0.05) was observed only for the superficial regions of Cancer B and D.

The EDTA gland isolation method may bias sampling to superficial tumor regions, which may be the oldest part of a tumor that progresses via clonal evolution (Fig 1). Laser capture microscopy (LCM) can sample multiple superficial, invasive and metastatic portions of the same CRC (Fig 6A). Only the BGN tag was used for analysis because its lower apparent error rate facilitates detection of recent clonal evolution. The degraded DNA in the fixed specimens and small numbers of genomes sampled by LCM hinders characterization of intragland PWD, but it is possible to compare intergland PWDs to search for focal regions of homogeneity created by recent clonal evolution. Consistent with the ability to measure tumor diversity with LCM, intergland PWDs were similar for Cancers A–E whether calculated from LCM or EDTA gland data (Fig S2).

With sequential stepwise progression, there should be a diversity gradient (superficial > invasive > metastases), whereas all regions should be similarly diverse if a tumor is essentially a single clonal expansion. Nine CRCs were examined (Fig 6B). Diversity was generally high, and none appeared to be recent clonal expansion because they all had average intergland PWDs greater than the clonal xenografts.

Intergland PWDs were similar between superficial and invasive portions of three Stage II cancers (B,C,E). For two of the six metastatic cancers (D and F), superficial, invasive, and metastatic regions were similarly diverse, including three different lymph node metastases of Cancer F. Significant diversity differences were present between four primary cancers and their metastases. Both the invasive and metastatic regions of Cancers H and I were significantly less diverse than their superficial cancer regions. For Cancer G, only the metastatic lesion was significantly less diverse. The metastasis of Cancer A was significantly more diverse than its primary.

To search for regional clonal evolution, intergland PWDs were compared with physical distances (Fig 6C). The superficial, invasive and metastatic regions of seven cancers appeared to be single clonal expansions because there were no significant changes in PWDs with physical distances. The superficial regions of Cancers B and D showed a significant increase in PWDs with physical distances, suggesting adjacent tumor areas were more related than distant areas. In summary, the diversity of different parts of the same cancer cannot be predicted, with examples of metastatic regions with intergland PWDs that were the same, greater, or smaller than their superficial regions. However, evidence of recent stepwise progression was lacking because all regions were relatively diverse (average PWDs greater than the clonal xenografts) and focal regional homogeneity was usually not detectable.

## Discussion

Tumor cells encounter and colonize many different microenvironments during tumorigenesis, and conceptually selection efficiently maximizes fitness to drive this progression. Clonal evolution depends on new driver mutations, which should be readily generated by the genomic “instability” thought to be present in many cancers [17]. However, recent CRC genome data demonstrate relatively low mutation frequencies (<1 per 100,000 bases), consistent with normal mutation and division rates [10]. Neutral passenger mutations predominate [6], [7], and therefore bona fide driver mutations may only rarely emerge in the relatively short intervals between transformation and tumor removal. If driver changes are rare, clonal evolution would be rare.

Without a measure of selection it is difficult to judge the roles of the numerous mutations and epigenetic changes found in cancer genomes. Selection efficiently optimizes fitness whenever and wherever opportunities arise, but a practical question is how much cells differ before selection intervenes. Although selection is difficult to measure, a selective sweep produces a bottleneck and loss of cellular diversity. Therefore, the diversity of hitchhiking passenger changes within a population (Fig 2) is a measure of selection [8]. A sensitive test for selection is the amount of hitchhiking variation within small cancer glands because fixation is faster in smaller populations [9]. The high passenger methylation pattern diversity measured in this study within and between small CRC gland fragments suggests selection is a weak force that typically lacks the minimum ability to sweep even nearby cells. This inferred lack of selective sweeps after transformation is consistent with the inability to readily identify additional metastatic driver mutations despite deep sequencing [2], [18]. A recent analysis of cancer genome data using a very different approach inferred that even driver mutations may confer relatively small selective advantages [19], which would also be consistent with the high passenger methylation diversity observed in cancer cell populations.

If selection is weak and a stepwise acquisition of new capabilities occurs infrequently, the first transformed cell may already produce well-adapted and versatile progeny with abilities to invade or metastasize [3]. Phenotypic progression after transformation would depend on phenotypic plasticity [4], [5], with invasion and metastasis from aberrant differentiation rather than the selection of new driver mutations. A single expansion is consistent with the similar diversities between glands regardless of physical distance in superficial, invasive, and metastatic lesions. The relatively high diversities of the CRC metastases imply relatively old populations, consistent with the early dissemination of tumor cells observed in experimental systems [20]. Significant diversity differences were sometimes observed between superficial, invasive, and metastatic regions of the same tumor. Such differences could represent stepwise selection, but could also arise without clonal evolution from different arrival times, with deeply invasive and metastatic regions colonized later in progression. Regional differences in mitotic rates could also produce differences, including situations where metastases are more diverse than their primary tumors. The high passenger methylation diversities in most CRCs and their metastases indicate relatively old and stable populations, with many divisions between transformation and surgery.

Without clonal evolution, present day tumor cells would form a single population with uncomplicated star-shaped ancestries and frequent long-lived lineages. The relative diversities within glands versus between glands (intragland to intergland PWD ratios) indicate how much remodeling or extinction occurs within glands. Simulations of cancer gland diversity suggested limited cancer cell extinction and were consistent with stem cell hierarchies with multiple long-lived CSC lineages per gland rather than extremely rare CSCs. CRCs are often resistant to chemotherapy [21], and greater numbers of long-lived lineages would more efficiently accumulate pre-existing therapy resistant variants.

Tumor evolution is commonly thought to increase fitness, but if the first transformed cell is already optimally “fit”, its progeny may suffer from progressive declines in fitness, an asexual reproduction phenomenon called Muller's Ratchet [22]. The limited but relatively ubiquitous extinction inferred in the CRCs may more represent loss of less fit variant cells (or background selection [8]) rather than dominance by more fit variants (Fig 2). Interestingly, passenger methylation pattern diversity and estimated numbers of CSCs were less in MMR deficient CRCs, where opportunities for selection would theoretically be greater because mutation rates are about 100 to 1,000-fold higher [16]. Potentially this lower diversity could reflect lower proliferation rates, although the intergland tag comparisons should help normalize mitotic ages between the MMR deficient and proficient CRCs. However, because mutations are more likely to be deleterious rather than advantageous, the lower passenger methylation pattern diversity in MMR deficient CRC glands may also represent negative selection with increased lineage extinction (fewer long-lived lineages) rather than positive selection and localized clonal evolution. This decreased fitness with higher mutation rates may help account for the better clinical outcomes for patients with MMR deficient CRCs [23]. A ratchet-like decline in fitness with time may also help explain why tumor growth progressively slows (Gompertzian growth [24]) rather than accelerates after transformation. The greater extinction with elevated mutation rates likely depends on the microenvironment because the Lovo cell line is MMR deficient [25] yet background selection was not as evident during xenograft formation in immunodeficient nude mice. These observations are consistent with a hypothesis that MMR deficient CRCs may have better outcomes because of an immune response to new antigens created by higher mutation rates [26].

Exactly how individual human tumors progress is uncertain because serial observations are impractical and unethical. Translation of traditional molecular phylogeny approaches to somatic cell populations can potentially reconstruct the pasts of individual human tumors [27], but many unknowns (including cell proliferation and death rates, and differences in rates and types of mutations) can confound analysis. Detailed ancestries are not possible with the current data, and many mechanisms may contribute to the high passenger methylation variations observed in cancer glands. Positive or negative background selection is likely to occur, but the high passenger methylation pattern diversities within and between cancer glands appear inconsistent with frequent or widespread clonal evolution sweeps that are commonly presumed to occur after transformation. Recent comparisons of copy number variations between single breast cancer cells from the same tumor also infer tumor populations emerge suddenly rather than gradually [28]. Recent cancer genome sequencing studies comparing somatic mutations from different parts of the same tumor infer evolution over many years, with many differences between metastases and primary pancreatic adenocarcinomas [29], [30]. The current approach is potentially complementary to sequence comparisons, being more suited to reconstruct more recent evolution over months instead of years due to higher inferred epigenetic replication error rates. Advances in sequencing technologies combined with defined topographical sampling of tumor populations should help reconstruct how individual human cancers evolve.

## Materials and Methods

### Ethics Statement

CRC samples were obtained in the course of routine clinical care from the Norris Comprehensive Cancer Center, with approval from our institutional review board (University of Southern California Health Sciences Campus Institutional Review Board, Proposal #HS-043078). The mouse xenograft studies were approved by our institutional review board (University of Southern California Institutional Animal Care and Use Committee, Protocol # 9606).

### Lovo studies

Limiting dilution was used for single cell cloning of the Lovo CRC cell line [13] into 96 well plates, verified by microscopy. Xenografts were initiated with subcutaneous injection of one million cells into nude (nu/nu) mice. On sacrifice, the xenografts were minced and small 2,000 to 10,000 fragments were isolated after stirring in an EDTA solution, as previously described for intestinal tissues [12]. DNA was extracted, bisulfite treated, and then amplified for the BGN and LOC tags. The PCR products were cloned into bacteria (TA-cloning kit, Invitrogen) and individual clones were sequenced.

### CRC studies

CRC samples were obtained in the course of routine clinical care from the Norris Comprehensive Cancer Center, with approval from our institutional review board. The 24 tags per gland sampling was performed on previously analyzed tumors (Cancers A–E are respectively Cancers 10, 3, 4, 12, and 5 in Ref 12). LCM was performed as previously described [12] on formalin-fixed microscope slides. The entire microdissected areas (about 1,000 to 2,000 cells) were subjected to bisulfite sequencing. Simulations of human CRC ancestries with different numbers of deterministic or probabilistic CSCs [12] were used to estimate CSC frequencies, and determine whether different parts of the same tumor (superficial, invasive, metastatic) were different (significance was when average regional PWDs were outside of 95% simulation intervals).

## Supporting Information

Figure S1 Methylation tags. **A.** Sequences of the LOC and BGN X-chromosomal tags. Primers are underlined and CpG sites are highlighted in red. **B.** Sample data, with 8 epialleles sampled from each specimen. The polyclonal specimens are more diverse (higher average PWDs) compared to the clonal culture or xenograft. Filled circles represent methylated CpG sites.
(PDF)

Figure S2 Intergland PWDs were similar with EDTA- (black) or LCM-sampling (red) for Cancers A–E, indicating the sampling approaches are equivalent. Only for Cancer B were the values significantly different (p<0.05, t-test).
(PDF)
