# Supplementary material for: High DNA Methylation Pattern Intratumoral Diversity Implies Weak Selection in Many Human Colorectal Cancers
Source: PLoS One. 2011 Jun 28;6(6):e21657. doi: 10.1371/journal.pone.0021657 (PMC3125304; doi:10.1371/journal.pone.0021657)
Supplement: Figure S1 — Methylation tags. A. Sequences of the LOC and BGN X-chromosomal tags. Primers are underlined and CpG sites are highlighted in red. B. Sample data, with 8 epialleles sampled from each specimen. The polyclonal specimens are more diverse (higher average PWDs) compared to the clonal culture or xenograft. Filled circles represent methylated CpG sites. (PDF) [file pone.0021657.s001.pdf]

A

BGN Tag 5’ TTTaggagtgagtagTtgTtttCGgtT**CG**T**CG**gaTaT
aT**CG**gaTagataga**CG**tg**CG**ga**CG**gTTTaTTaTTTTagTT**CG**T
TaaTtagtTagTTtg**CG**TTtgg**CG**TTtTTTTtTtTTaggtagggTtggT

LOC Tag 5’ gttgtgggaTaggtgTaggaaTtTtaggTTtggTttTatgtg
CGCGaa**CGCG**tagTTagggatg**CG**Tt**CG**atgtggt**CG**tggtgTag
CGtTagg**CG**TTtTaggTTT**CGCG**gTaggaag**CG**gggTaCGtgT
aTTagT**CG**gttgtaggaTaggtTaaggg

B

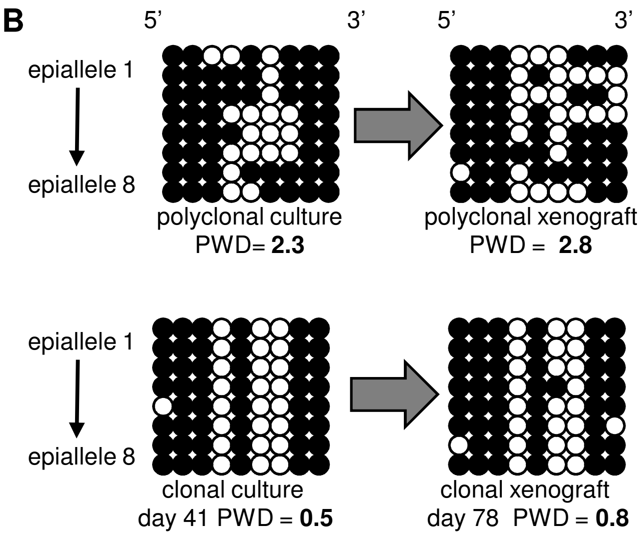

SOM Figure 1)
A. Sequences of the LOC and BGN X-chromosomal tags. Primers are underlined and CpG sites are highlighted in red.
B. Sample data, with 8 epialleles sampled from each specimen. The polyclonal specimens are more diverse (higher average PWDs) compared to the clonal culture or xenograft. Filled circles represent methylated CpG sites.
