# Supplementary material for: High DNA Methylation Pattern Intratumoral Diversity Implies Weak Selection in Many Human Colorectal Cancers
Source: PLoS One. 2011 Jun 28;6(6):e21657. doi: 10.1371/journal.pone.0021657 (PMC3125304; doi:10.1371/journal.pone.0021657)
Supplement: Figure S2 — Intergland PWDs were similar with EDTA- (black) or LCM-sampling (red) for Cancers A–E, indicating the sampling approaches are equivalent. Only for Cancer B were the values significantly different (p<0.05, t-test). (PDF) [file pone.0021657.s002.pdf]

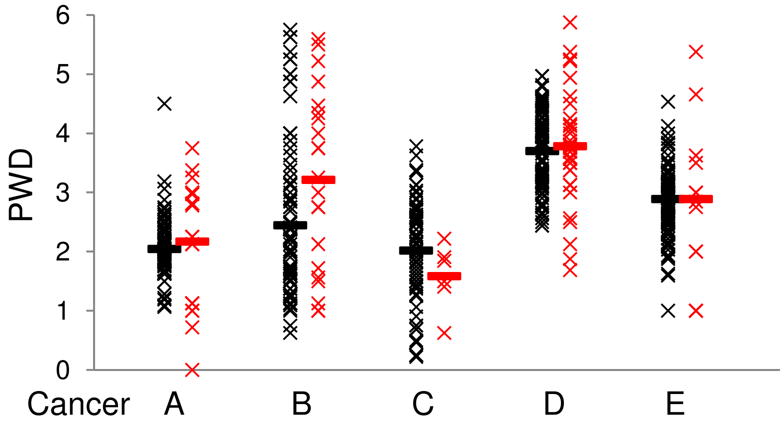

SOM Figure 2) Intergland PWDs were similar with EDTA- (black) or LCM-sampling (red) for Cancers A-E, indicating the sampling approaches are equivalent. Only for Cancer B were the values significantly different ($p < 0.05$, t-test).
